# Psychotropic Polypharmacy Among Youths Enrolled in Medicaid

**DOI:** 10.1001/jamanetworkopen.2023.56404

**Published:** 2024-02-16

**Authors:** Yueh-Yi Chiang, Alejandro Amill-Rosario, Phuong Tran, Susan dosReis

**Affiliations:** 1 Department of Practice, Sciences, and Health Outcomes Research, University of Maryland School of Pharmacy, Baltimore

## Abstract

This cross-sectional study aims to identify temporal changes and characteristics associated with psychotropic polypharmacy among youths aged 17 years or younger who were enrolled in Medicaid in Maryland.

## Introduction

Concomitant use of medications for attention-deficit/hyperactivity disorder (ADHD), antipsychotics, mood-stabilizing anticonvulsants, and antidepressants is referred to as psychotropic polypharmacy.^1^ Over the past 2 decades, psychotropic polypharmacy in youths increased, raising safety concerns.^2,3,4^ Our goal was to examine trends from 2015 to 2020 in psychotropic polypharmacy among youths aged 17 years or younger who were enrolled in Medicaid to identify temporal changes and characteristics associated with psychotropic polypharmacy.

## Methods

The cross-sectional study was approved by the University of Maryland institutional review board and followed the Strengthening the Reporting of Observational Studies in Epidemiology (STROBE) reporting guideline. Informed consent was waived because data were deidentified.

This is a sequential, annual, cross-sectional study using Medicaid eligibility files and fee-for-service and managed care medical encounter claims from 2015 to 2020 from a single US state. For each annual cohort, we included youths who were 17 years or younger, had received at least 1 pharmacy claim for psychotropic medication, and had 90 days or more of continuous Medicaid enrollment. We created 4 mutually exclusive Medicaid eligibility groups in each annual cohort: (1) youths with low income, (2) youths enrolled in Children’s Health Program (CHP), (3) youths in foster care, and (4) youths with disabilities. Additional information regarding the methods can be found in the eMethods in Supplement 1. Using the American Hospital Formulary Service Pharmacologic Therapeutic Classification System, we classified psychotropic medications into 6 therapeutic classes: antipsychotics, ADHD medications, mood-stabilizing anticonvulsants, antidepressants, anxiolytics, and sedatives. Use of 3 or more different psychotropic classes that overlapped for 90 consecutive days or longer in each study year defined psychotropic polypharmacy.^1^ We allowed no more than a 15-day gap between prescription fills within the 90-day period. Annual psychotropic polypharmacy prevalence was defined as the proportion of youths who had at least 1 polypharmacy episode per 100 youths with any psychotropic use. A multivariable logistic regression model, using all study years, estimated the odds of psychotropic polypharmacy (dependent variable) among psychotropic users. Independent variables included study year (continuous), age, sex, race, region, Medicaid eligibility group, COVID-19, and mental health disorders. The race and ethnicity categories included in this study were American Indian, Asian, Black, Hispanic, Native Hawaiian, White, and all other races not listed in one of the predefined categories. Information regarding race and ethnicity was extracted from the Medicaid demographic summary file. We used generalized estimating equations for robust variance estimators to account for nonindependence of observations given that a youth may be in multiple study years. A supplemental analysis modeled categorical year (reference year, 2015) to identify which years contributed to significant psychotropic polypharmacy changes. Significance levels were set at *P* < .05 for 2-tailed tests. All analyses were performed using SAS Studio version 9.4 (SAS Institute). Data were analyzed from January to December 2023.

## Results

Across all years, 126 972 unique youths met the inclusion criteria. Psychotropic polypharmacy prevalence among youths who used psychotropics increased from 2259 of 53 569 youths (4.2%) in 2015 to 2334 of 50 806 youths (4.6%) in 2020. The 2015 to 2020 increase in psychotropic polypharmacy prevalence was observed for those with Medicaid eligibility from foster care (414 of 3824 [10.8%] and 387 of 3420 [11.3%]), CHP (225 of 10 354 [2.2%] and 222 of 7974 [2.8%]), and being from a low-income household (648 of 30 222 [2.1%] and 883 of 31 172 [2.8%]) (Figure). The adjusted odds ratios (AORs) of psychotropic polypharmacy for the year was 1.04 (95% CI, 1.02-1.06), a 4% increase in the odds of psychotropic polypharmacy per year. The supplemental analysis revealed a significant increase in 2019 and 2020 relative to 2015. Psychotropic polypharmacy was significantly more likely among youths who were disabled (AOR, 3.68; 95% CI, 3.34-4.05) or in foster care (AOR, 3.31; 95% CI, 2.93-3.74) relative to youths in the low-income group. Individuals aged 10 to 14 years (AOR, 1.94; 95% CI, 1.80-2.10) and 15 to 17 years (AOR, 2.41; 95% CI, 2.22-2.61) had significantly higher odds of psychotropic polypharmacy than those who were younger than 10 years. Black individuals (AOR, 0.47; 95% CI, 0.43-0.51) or individuals who identified as other races (including individuals identifying as American Indian, Asian, Hispanic, or Pacific Islander, or other races) (AOR, 0.54; 95% CI, 0.50-0.59) had significantly lower odds of psychotropic polypharmacy than White individuals (Table).

**Figure.**
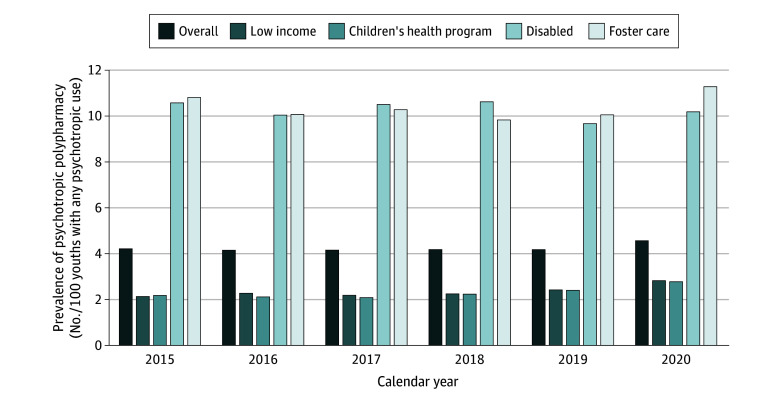
Prevalence of Psychotropic Polypharmacy Among Youth Enrolled in Medicaid With Any Psychotropic Use by Eligibility Group, 2015 to 2020 In calculating the annual polypharmacy prevalence, the numerator was the number of youths who had at least 1 polypharmacy episode of 90 or more consecutive days in the study year. The denominator was the number of youths who had at least 1 pharmacy claim for a psychotropic medication and 90 days or more of continuous Medicaid enrollment during the same study year.

**Table.** Characteristics Associated With Psychotropic Polypharmacy Among Youths Enrolled in Medicaid With Any Psychotropic Use

Study cohort variables	Participants, No. (%) (N = 126 972)	AOR (95% CI)	*P* value
Year (continuous variable)	NA	1.04 (1.02-1.06)	<.001
Age, y			
0 to 9	55 449 (43.7)	1 [Reference]	NA
10 to 14	43 956 (34.6)	1.94 (1.80-2.10)	<.001
15 to 17	27 567 (21.7)	2.41 (2.22-2.61)	<.001
Sex			
Female	54 759 (43.1)	1 [Reference]	NA
Male	72 213 (56.9)	1.06 (1.00-1.13)	.06
Race^a^			
Black	42 725 (33.6)	0.47 (0.43-0.51)	<.001
White	45 643 (35.9)	1 [Reference]	NA
Other^b^	38 604 (30.4)	0.54 (0.50-0.59)	<.001
Region			
Western	11 163 (8.8)	1 [Reference]	NA
Capital	30 369 (23.9)	0.69 (0.61-0.78)	<.001
Central	60 867 (47.9)	0.75 (0.68-0.83)	<.001
Southern	7117 (5.6)	0.62 (0.52-0.74)	<.001
Eastern shore	17 456 (13.8)	0.80 (0.71-0.90)	<.001
Eligibility group			
Low income	79 064 (62.3)	1 [Reference]	NA
CHP	25 425 (20.0)	0.93 (0.85-1.03)	0.13
Disabled	15 114 (11.9)	3.68 (3.34-4.05)	<.001
Foster care	7369 (5.8)	3.31 (2.93-3.74)	<.001
COVID-19			
Year 2015-2019	NA	1 [Reference]	NA
Year 2020	NA	1.18 (1.10-1.25)	<.001
Mental health disorders^c^			
Without evidence of mental health disorders	NA^d^	1 [Reference]	NA
With evidence of mental health disorders	NA^d^	2.84 (1.05-2.57)	<.001

Abbreviations: AOR, adjusted odds ratio; CHP, Children’s Health Program; NA, not applicable.

^a^ Race was grouped into Black individuals, White individuals, and others to avoid small cell sizes.

^b^ Other race includes individuals who identified as American Indian, Asian, Hispanic, Pacific Islander, and all other races that are not listed in a predefined category.

^c^ Evidence of mental health disorders included anxiety, bipolar disorder, depression, posttraumatic stress disorder, attention-deficit/hyperactivity disorder, conduct disorder, oppositional defiant disorder, autism, substance abuse disorder, and adjustment disorder.

^d^ Due to the dynamic changes in the prevalence of such characteristics per year, they may not be accurately represented in a table with all years concatenated. Results for the prevalence of the characteristics stratified by year are available upon request from the first author.

## Discussion

In this cross-sectional study, we observed a 4% increased odds of psychotropic polypharmacy per year from 2015 to 2020, indicating growing concomitant use of multiple psychotropic classes. Among youths enrolled in Medicaid with any psychotropic use, individuals who were disabled or in foster care were significantly more likely than individuals with low income to receive 3 or more psychotropic classes overlapping for 90 days or more. Factors such as complex medical conditions, early-life trauma, and fragmented care may have contributed to these findings.^5^ This study was limited by the focus on youths enrolled in Medicaid in a single US state, which limits the generalizability of our findings to other states, populations, or health care systems. The findings emphasize the importance of monitoring the use of psychotropic combinations, particularly among vulnerable populations, such as youths enrolled in Medicaid who have a disability or are in foster care.

## Notes

Supplement 1.
eMethods.

Supplement 2.
Data Sharing Statement
