# Supplementary material for: Psychotropic Polypharmacy Among Youths Enrolled in Medicaid
Source: JAMA Netw Open. 2024 Feb 16;7(2):e2356404. doi: 10.1001/jamanetworkopen.2023.56404 (PMC10873764; doi:10.1001/jamanetworkopen.2023.56404)
Supplement: Supplement 1. — eMethods. [file jamanetwopen-e2356404-s001.pdf]

Supplemental Online Content

Chiang Y, Amill-Rosario A, Tran P, dosReis S. Psychotropic polypharmacy among youth enrolled in Medicaid. *JAMA Netw Open.* 2024;7(2):e2356404. doi:10.1001/jamanetworkopen.2023.56404

eMethods.

This supplemental material has been provided by the authors to give readers additional information about their work.

© 2024 Chiang Y et al. *JAMA Network Open.*

eMethods.

Medicaid Eligibility Group

Four Medicaid eligibility groups identified in each annual cohort were: 1) low-income, i.e., family income is at or below 200% of the federal poverty level (FPL); 2) Children's Health Program (CHP), i.e., family income is between 200-400% of FPL; 3) foster care, i.e., those who are removed from their biological parents; and 4) disabled, i.e., those who have a qualifying cognitive, physical, or developmental disability. Since individuals may change Medicaid eligibility groups within a year, we applied a hierarchical approach to create four mutually exclusive Medicaid eligibility groups: 1) disabled, 2) foster care, 3) CHP, and 4) low-income. All those assigned to a group lower in the hierarchy could not have any evidence of eligibility in a group higher in the hierarchy (i.e., foster care assigned could not have any evidence of disabled Medicaid eligibility).

© 2024 Chiang Y et al. *JAMA Network Open.*
