# Supplementary material for: Psychotropic Polypharmacy Among Youths Enrolled in Medicaid
Source: JAMA Netw Open. 2024 Feb 16;7(2):e2356404. doi: 10.1001/jamanetworkopen.2023.56404 (PMC10873764; doi:10.1001/jamanetworkopen.2023.56404)
Supplement: Supplement 2. — Data Sharing Statement [file jamanetwopen-e2356404-s002.pdf]

Data Sharing Statement

Chiang. Psychotropic Polypharmacy Among Youth Enrolled in Medicaid. *JAMA Netw Open*. Published February 16, 2024. doi:10.1001/jamanetworkopen.2023.56404

Data
Data available: No

Additional Information
Explanation for why data not available: The data agreement did not allow us to share the data; a data dictionary defining each field in the dataset will be made available upon request.
